# The diversity and consistency of what and when people eat

**DOI:** 10.21203/rs.3.rs-6149642/v1

**Published:** 2025-08-05

**Authors:** Tyler Tran, Emily Nicole Manoogian, Zhaoyi Joey Hou, Shweta Varshney, Jialu Sui, Kyla L. Laing, Jason Fleischer, Satchidananda Panda

**Affiliations:** 1. The Salk Institute for Biological Studies, La Jolla, CA 92037; 2. Department of Cognitive Science, University of California San Diego, La Jolla, CA, USA

## Abstract

The timing and types of food people eat, along with daily fluctuations in in these parameters, are important factors influencing health and well-being. However, there is limited data on how eating patterns remain consistent over multiple days. To address this gap, we conducted an exploratory, observational analysis of over 20,000 adults, who recorded more than 2.5 million food logs collected over two weeks via the myCircadianClock app. Our analysis reveals significant variability in food timing and diversity. The time window during which 95% of food and beverages were consumed during the 2 weeks ranged from 10 hours 54 minutes for the lowest decile to over 16 hours for the highest. The median number of unique food and beverage items consumed over the two weeks varied from 20 to 86, but only a subset of these items was consistently eaten on multiple days. Many common foods were regularly consumed at specific times of the day, and factors like age, gender, and work schedules influenced both eating patterns and food choices. These findings provide a foundation for utilizing longitudinal food records in nutrition and lifestyle research to enhance our understanding of human behavior and health.

## Introduction

Understanding how the quantity (total energy intake) and quality (macronutrient composition) of dietary intake relate to population health has long been a central focus of public health research and policy. The 7-day food list recall, originally adopted by the U.S. Department of Agriculture in the early 20th century ^1^, laid the foundation for the development of the 24-hour dietary recall (24HDR). Among the methods available for capturing dietary behaviors at scale, the 24HDR has emerged as a widely accepted and pragmatic tool ^2,3^. Since its formal inclusion in the National Health and Nutrition Examination Survey (NHANES) beginning in 1971–1974, the 24HDR has served as a cornerstone of nutritional surveillance in the United States, enabling population-level assessments of dietary exposures and their associations with health outcomes ^4,5^ —albeit with certain limitations ^6^.

Over the past five decades, considerable methodological refinement has improved the reliability and granularity of the 24HDR. Recognizing that dietary intake varies not only day-to-day but also between weekdays and weekends—often due to social and occupational factors—NHANES adopted protocols to collect 24HDRs from both weekday and weekend days ^7^. Two or three 24HDRs also better report energy intake than a single 24HDR ^8^ and repeat 24HDR along with appropriate statistical methods also improved reporting of nutrient intake by subpopulations ^9,10^. To reduce underreporting and enhance recall accuracy, the U.S. Department of Agriculture developed the five-step Automated Multiple-Pass Method (AMPM), which structures recall by associating food intake with defined eating occasions (e.g., breakfast, snack, dinner) and their timing ^11,12^. This time-anchoring prompt aids memory and has expanded understanding of the frequency and content of eating events across demographic subgroups. The use of 24HDR in NHANES, the UK Biobank, and other population-based cohorts has solidified its role as a core instrument for nutritional epidemiology as well as in prospective studies.

Although originally designed to capture energy and nutrient intake, the 24HDR has also evolved to assess broader dimensions of diet quality—including food group composition and dietary diversity ^13–15^. Historically, dietary variety was positively associated with improved nutrient adequacy, reduced mortality, and lower risk of chronic diseases, leading to its inclusion in U.S. dietary guidelines for much of the 20th century. Yet in recent decades, this narrative has been challenged ^16,17^. Proliferation of foods—over 12,000 new products entering the U.S. market annually—has reframed dietary variety as a potential driver of overeating and metabolic dysregulation ^18^. Meanwhile, consumer behavior research suggests that food choices may be less habitual than what is interpreted from epidemiological data ^19^, raising concerns about the validity of single-day dietary assessments in capturing long-term eating patterns.

Emerging evidence from circadian biology now suggests that when food is consumed—its timing relative to the sleep-wake cycle and circadian physiology—plays a critical role in metabolic regulation and disease risk ^20^. Daily fluctuations in digestion, nutrient absorption, insulin sensitivity, and glucose metabolism are all governed by circadian rhythms ^21,22^. Consequently, features of an individual’s eating pattern—such as the duration of the daily eating window, its timing relative to sleep, and day-to-day variability—may influence cardiometabolic outcomes ^23–25^. These insights have prompted a wave of retrospective analyses using 24HDR data to infer temporal eating patterns and their associations with obesity, diabetes, and cardiovascular disease.

However, the utility of 24HDR for this purpose remains debated. In NHANES, time-of-day data are used as memory aids rather than validated time stamps, and independent studies have found only modest concordance (r = 0.15–0.45) between 24HDR-reported eating times and contemporaneous food diaries ^26^. Nevertheless, several analyses assume that a single 24HDR reflects habitual eating windows, often generalizing weekday or weekend patterns across extended periods. As a result, estimates of the average American adult’s eating window vary widely—from 10 to 13 hours—depending on the study ^27–29^. Reports have also reached conflicting conclusions on whether shorter eating windows confer cardiometabolic benefit or pose risks, such as increased cardiovascular mortality, particularly when achieved through prolonged morning or evening fasting ^30–32^.

The limitations of cross-sectional recall data have become even more apparent considering prospective time-restricted eating (TRE) trials. These studies suggest that compressing the daily eating window to 6–10 hours can improve metabolic outcomes ^33^. Yet questions remain: Do TRE benefits arise from changes in nutrient timing, food choices, caloric intake, or circadian alignment? Studies have shown that TRE can inadvertently reduce energy intake, improve nutrition quality, and as different foods are preferred at different times of the day, early or late TRE cohorts can also differ in their food choices, potentially confounding associations between meal timing and health outcomes.

Addressing these complex questions requires innovation in dietary assessment methodologies that go beyond energy and nutrient intake to capture longitudinal eating behaviors, including timing, frequency, and consistency ^34^. Web-based tools like ASA24 and UK WebQ have expanded the reach of traditional 24HDR, but they remain constrained by respondent burden and limited time resolution. Smartphone adoption, which now exceeds 90% among U.S. adults, offers new opportunities to capture dietary habits in real time and real-world settings. However, most of the smartphone apps are developed to promote nutrition quality and quantity ^35^.

To meet this challenge, we developed the myCircadianClock (mCC) smartphone application, which enables users to log dietary intake using natural language or images, with automatic timestamping. To minimize food-logging-induced changes in dietary habits, the users are blinded to their data for the first 2 weeks ^36^. Following initial validation in smaller cohorts ^37^, the mCC app has been deployed in pilot and randomized controlled trials to monitor and modulate eating patterns ^38–45^. To explore real-world feasibility and scalability, we also launched a public version of the app, allowing over 21,000 adults (age ≥18y) to log their habitual dietary patterns over a 2-week period.

In this study, we present an analysis of these self-reported data, focusing on the consistency of eating windows, variability in food choices, and demographic influences such as age, gender, and occupation. Our findings underscore the heterogeneity of eating behaviors and demonstrate the feasibility of leveraging mobile technology to capture the temporal dimensions of diet at scale. These insights offer a foundation for more nuanced and temporally aware nutrition research and interventions.

## Results.

### Participants and dietary records.

We analyzed food and beverage (f/b) logs from adults (age >18y) who digitally consented to use the myCircadianClock app ^36^ to record all their ingestive behavior for 2 weeks. To minimize the potential effects of diet self-monitoring on dietary habits, ^46^, the users could not review their logs and were not given any feedback or advice about their diet quality, quantity, and timing during this 2 weeks period. They received a daily reminder to record their dietary intake. Participants were asked to log all their ingestion events in real time with a simple log in natural language and encouraged to take a picture of the food or beverage. The log was timestamped by the phone with a “log-time” and transmitted to the server where it is recorded with a timestamp of the server (server-time). If the participant forgot to log in real time, s/he could log the food and enter an approximate time of consumption (log-time). In case of poor or no internet or Wi-Fi signal, the log waited in the phone until transmission to the server was possible. The time when the log reached the server was also recorded as server time. The time difference between the log time and server time reflected whether the food record was recorded in real-time or was delayed due to retrospective user logging or delay in transmission from smartphone to server. We estimated 93.54% of logs were recorded real-time on the smartphone device.

**Fig. 1.**
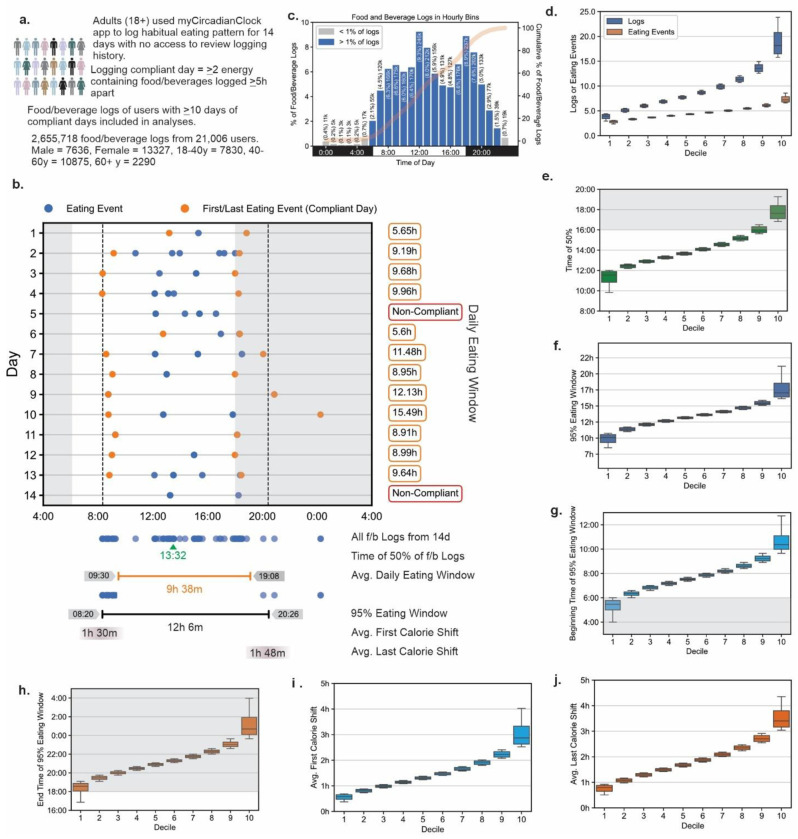
Eating pattern from fourteen days of food records. **a**. Schematic of data collection from the myCircadianClock smartphone app. **b**. Representative two weeks of food and beverage record from a user and various parameters describing eating pattern discussed in the manuscript. **c**. Frequency distribution of food and beverage logs recorded in hourly bins. **d**. number of food logs and eating occasions (groupings of food logs), **e**. median time of all food logs (TF50) **f**. length of 95% eating window (eating window), **g.** start time of eating window **h**. end time of eating window **i**. day-to-day first eating event shift **j**. day-to-day last eating event shift from each decile (~2100 users/decile bin) bin of users. For d-j the deciles were calculated independently for the respective parameters. Plot whiskers range from 2.5^th^ to 97.5^th^ percentiles

Reliability of timing of breakfast and dinner increases with repeated 24 h recalls and reaches >75% after at least nine 24HDR ^47^. Prior studies using the same app found individuals who consent to log all their ingestive behavior for two weeks on average log for at least 10 days with >2 calorie-containing food or beverages per day, >5 hours (h) apart ^38,41^. Using this criteria, there were 21,006 adults (male = 7621, female = 13321, demographic data in **Table S1**) who recorded 10-14 days of food and beverage records (total number of f/b logs= 2,655,718; the number of f/b records/person mean 126.43, sd 62.91; male mean 122.38, sd 62.64; female mean 128.71, sd 62.94), used for further analyses (Fig 1a, 1b).

Prior sleep studies have shown many people can go to bed after midnight ^48^ and human activity reaches a trough between 2 am and 4 am ^49^, therefore 04:00 to 03:59:59 the next day was considered the beginning and end of the 24h day. The frequency of f/b logging (individual entries), as seen earlier with smaller cohorts ^37,39,40,50^, showed a daily rhythm with low consumption (<1% of all logs/ hourly bin) between 23:00 and 05:00 and two peaks at 12:00 and 18:00 local time (9.29% and 8.93% of all records respectively) (Fig 1c). 20.35%, 40.58%, 35.95%, and 3.12% f/b records were logged in the 1st (04:00-10:00), 2nd (10:00-16:00), 3rd (16:00-22:00), and 4th (22:00-04:00) quartile of the 24h day (Fig.1c, **Table S2**). Food and beverage entries logged within 15 minutes (m) of each other were considered an eating event ^37,50^. The bottom decile (~2100 people/decile bin) logged 4.57 records within 3.08 eating events per day, while the top decile logged over 15.29 records within 6.50 eating events (Fig 1d, **Table S3**). This population level results are comparable to what is observed in 1-5 days of food records from other studies such as NHANES data ^51^.

### Daily Eating pattern.

A raster plot of all energy containing food logs over 14 days from a randomly picked participant (Fig 1b) shows the eating pattern is not consistent and can be described by various metrices. We used multiple metrics to assess daily eating patterns, each providing a unique aspect of eating pattern of each participant (**Table S4 and S5**). The time by which 50% of f/b consumption has occurred (TF50) is one of the measures for distinguishing early vs. late eaters; early eaters reach this median time earlier than late eaters ^52,53^. The TF50 of all f/b records for each user ranged from 12:07 for the earliest decile to 16:40 for the latest decile and median at 13:52 (Fig 1e, **Fig S1a-b**). However, the TF50 does not reflect the width of the window of time when people eat. The average daily eating window (the mean time between the first and last caloric intake of each compliant day, Figure 1b), ranged from 8h 32 m for the first decile to 12h 43m for the last decile with a mean of 10h 40m (sd 101m) (**Fig S1b-c**). However, due to the day-to-day variation in eating time including variation in the first and last eating event, ~25% of f/b logs were recorded outside the average eating window (**Fig. S1d**) demonstrating the limitation of this metric. Another measure of eating window that captures this day-today variation of eating times is the 95% eating window ^37^, which is the time between the 2.5%tile of all f/b logs (i.e. excluding a few exceptionally early f/b logs) and 97.5%tile of f/b log time (i.e. excluding a few exceptionally late f/b logs) over multiple days. The mean 95% eating window was 13h 29m (sd 134m) ranging from 10h 54m (bottom decile) to 16h 0m (top decile) (Fig. 1f, **Fig. S1f**). The mean beginning of the 95% eating window was 07:47, ranging from 06:00 (bottom decile) to 09:39 (top decile) (Fig. 1g, **Fig S1g**). The mean end of the 95% eating window was 21:16 ranging from 19:05 (bottom decile) to 23:39 (top decile) (Fig. 1h, **Fig S1h**). As 95% eating window, by definition, excludes only 5% of f/b logs, while the average eating window, on average, excludes 25% of f/b records, for the rest of the manuscript, we will use 95% window as “eating window”.

Day-to-day variation in the timing of the first eating event contributed to weight change in the CALERIE-II study ^54^. For each participant we calculate the difference between the time of an eating event (e.g., first eating event) on successive days where the participant is compliant with logging criteria. A participant’s day-to-day shift is the absolute value of this difference across the 14-day period. The first caloric intake shifted a mean of 1h 31m (sd 46m) between successive days across all participants (bottom decile 0h 42m and top decile 2h 28m) (Fig1i. **Fig S1i**), and their last caloric intake shifted a mean of 1h 53m (sd 50m) (bottom decile 0h 57m and top decile 2h 59m) between days (Fig1j. **Fig S1j**). Participants were more regular in the timing of their first calorie intake (26.1% had < 1h mean first calorie shift between successive days) than their last calorie intake (11.9% had <1h mean last calorie shift between successive days). Only 5.2% of users were consistent enough in the timing of their daily eating patterns that both first calorie and last calorie mean shifts were <1h (**Fig S2**). To test any relation between eating window and consistent timing of first or last calorie intake, we calculated the average first and last calorie shift for each percentile of eating window. We found, those with a shorter eating window were more likely to have a consistent eating pattern with first- and last-calorie shift <1h (**Fig S3**).

**Fig. 2.**
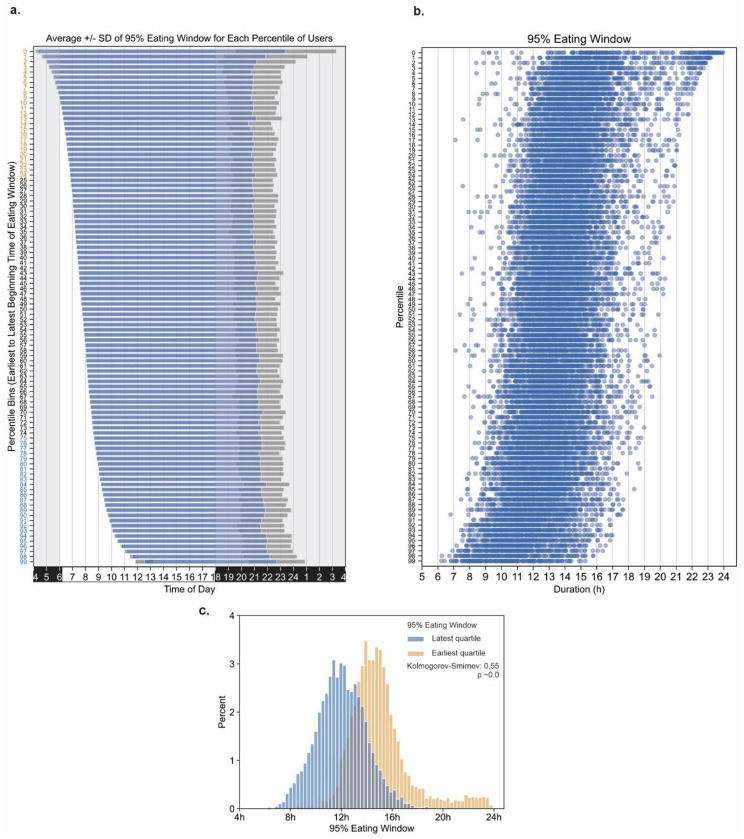
Relation between the start time of eating window with the length of eating window. **a**. Mean (+/− sd in shaded grey) of eating window for each percentile of users (~210/percentile bin). Bins are sorted by eating window start time. **b**. The eating windows of all individuals in each percentile bin are shown. Each data point represents the eating window of an individual; lower percentiles indicate earlier eating window start times. **c**. The distribution of eating window in each quartile of start time of eating window shows early eaters are likely to have a longer eating window.

Next, we tested whether when a person starts eating has any effect on the length of eating window. We ordered the eating window of all participants based on the start time of their eating window from earliest to latest and binned them in each percentile (100 bins; each bin containing ~210 participants) (Fig 2 a,b). The earliest percentile started their eating window at an average of 04:20 (std 10 m) and had an eating window of 19h 4m (sd 3h 55m). The latest percentile started their eating window at 12:32 (sd 42m) and had an average eating window of 10h 11m (sd 2h 1 m). Similarly, those who begin early (first decile; eating window start time between 04:00 and 6:00) are also likely to have a longer eating window (mean 15h 54m sd 172 m), while those in the top decile or those who start eating later (eating window start time between 09:39 and 18:07) were likely to report a shorter eating window (mean 11h 16m sd 118 m, Mann-Whitney U=4114091.50 p≅0.0 ) (Fig 2).

### Eating patterns are shaped by work schedule, age, and gender.

Next, we tested whether age, sex, or work type affected eating pattern. The absence of any effect would result in equivalent number of participants in each decile groups for eating window, start or end of eating window, TF50 and first meal or last meal shifts. Older participants were more likely to have a shorter eating window and finish their eating window earlier than the younger participants (**Table S6**). We found the shift workers were more likely to have irregular eating patterns marked by longer eating window, and larger first- or last-meal shift.

**Figure 3.**
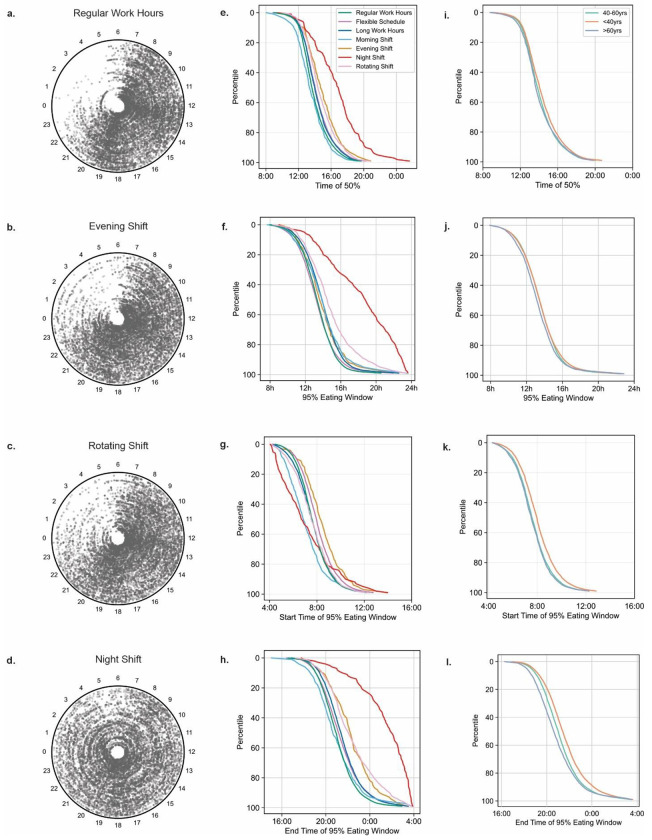
Effects of work schedule, sex and age on eating patterns. Polar (clock-style) plots of food and beverage logs for 150 randomly selected individuals who self-identified their work schedule as **a**. regular hours, **b**. evening shift, **c**. rotating shift, or **d**. night shift. Angle around the circle corresponds to clock time and each concentric circle represents one user. Each point represents the timing of a food log; denser regions indicate peak eating times. **e**. mean TF50 values arranged from earliest (top) to latest (bottom) of users from each work schedule shows night shift workers have remarkably late TF50. Percentile rank of **f**. eating window, **g**. beginning-. and **h**. end- of eating window of different work schedules. Percentile rank of **i**. TF50, **j**. average eating window, **k**. beginning of eating window and **l**. end of eating window of different age groups.

We further explored whether different types of shiftwork and whether self-described flexible schedules or long work hours affect eating patterns. In visual display of all eating occasions of randomly picked 150 participants of different work types, the eating occasions of night shift workers were more dispersed throughout the 24h day than that of regular hours workers (Fig 3a–d, **Fig S4**). The morning shift workers reached their median f/b log time earliest (mean 13:30, sd 116m) followed by regular work hours (mean 13:54, sd 107m), long work hours (mean 14:11, sd 109m), flexible schedules (mean 14:14, sd 114m), rotating shifts (mean 14:38, sd 118m), evening shifts (mean 14:51, sd 130m), and night shifts (mean 16:39, sd 182m) (Fig. 3e, **Fig S4, Table S7**). The eating window and the end of the eating window also differed significantly between some of the groups (Fig 3f–h and **Table S7**). The 95% eating window of self-described flexible workers was close to that of the regular schedule workers. The night shift workers had the longest 95% eating window (mean 18h 4m, sd 234m), largest mean day-to-day shift in first eating events (2h 58m, sd 98m) and last eating events (mean 2h 49m, sd 72m) and they were significantly different (**Table S7-10**) from that of regular schedule workers (95% eating window mean 13h 19m, sd 118m; first eating event shift mean 1h 26m, sd 41m; mean last eating event shift mean 1h 50m, sd 47m), who exhibited shorter and more stable eating windows than all shift worker groups.

The daily pattern of sleep and wakefulness is also known to change across age ^55^, albeit modestly as compared to shift work, which in turn influences eating event timing ^56^. Accordingly, we found the younger individuals started their first eating event later (<40y old; n= 7830; mean beginning of the 95% window 08:01 sd 91m) vs. older individuals (>60y old; n=2290; mean beginning of the 95% window 07:34 sd 85m), Younger individuals also reached their TF50 later (mean 14:16 sd 116m vs. 13:57 sd 115m) and had a longer eating window (mean 13h 33m sd 137m vs. 13h 16m sd 137m) than older individuals (Fig 3i–l and **Table S11**). The middle age group (40-60y, n= 10875) was intermediate in many of these measures (mean eating window 13h 30m sd 131m; mean start of eating window 07:39 sd 86m; mean TF50 14:02 sd 110m). However, the first eating event shift did not change remarkably between the young, middle aged or older age groups (mean 1h 32m sd 48m, 1h 30m sd 44m and 1h 31m sd 44m, respectively). The last eating event shift reduced modestly from young to older age groups (mean young 1h 55m sd 49m, middle-aged 1h 53m sd 50m, old 1h 49m sd 50m). Within each of these age brackets, the males and females also showed some differences (**Table S11**).

### What people eat

**Figure 4.**
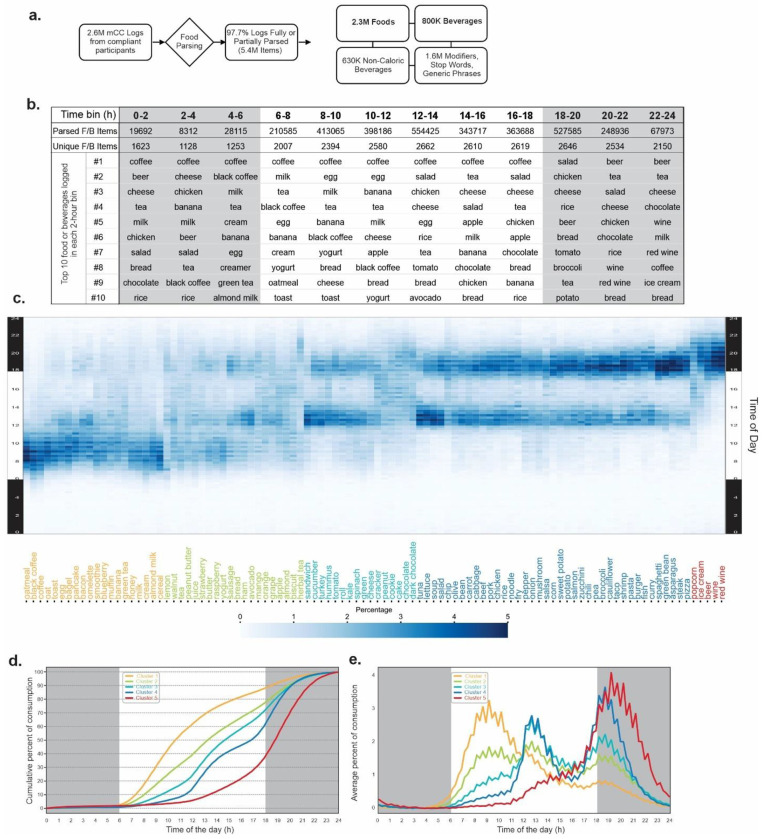
Food preference as a function of time-of-day. (a) Schematics of parsing food and beverage logs to individual food and beverage items. (b) Diversity and popularity of food and beverages in each 2h bin. The top ten most popular items in each bin are shown in rank order. The total number of items in each bin and number of unique items in each bin are shown. (c) Heatmap of hierarchically clustered top 100 food and beverage items and their timing of consumption in 15m bins over 24 h. The time of day (Y-axis) and the names of the items (X-axis) color-coded for the respective cluster. (d) The timing of consumption of items in five different clusters in 15m bins show distinct consumption patterns where cluster 1 is mostly preferred at breakfast, cluster 2 is throughout the day, Clusters 3 and 4 are preferred at lunch and dinner and cluster 5 is largely after dinner. (e) Cumulative consumption of items in five clusters is shown.

Participants used natural language in their f/b logs to describe food and beverages consumed. Therefore, we used food and beverage names to analyze what people eat, as they are more relatable in real life. The f/b logs recorded in natural language were heterogeneous in content and description; one log may describe only one food item (e.g. warm muffin), while some may contain multiple items (Latte, croissant and leftover pizza). Each f/b log was parsed by a custom parsing pipeline to extract food or beverage names and excluded generic terms (e.g., breakfast, lunch, dinner, Chinese food, etc.) or stop-words (e.g., the, a, etc.) and some modifiers (e.g., leftover, warm, grilled, spicy, etc.). As has been done in other studies such as re-analyses of NHANES records ^57^, similar foods or beverages were also assigned one name (e.g. bread, whole wheat bread, whole grain bread were grouped as bread). This yielded 2227 foods and 530 beverages in the library. The logs from 21,006 users yielded 2.3 million foods and 800 thousand beverages that matched a common name in the library (Fig 4a). 97.7% of logs contained items that could be matched to common names (Fig 4a). We assessed how ~>3million parse items representing 2757 foods and beverages were consumed across 24h day.

As expected, for the entire cohort, the diverse types of food logged at different times of the day varied. Only 8312 parsed items representing 1128 unique f/b were logged between 02:00-04:00, while 554,425 items (2662 unique f/b) were logged between 12:00-14:00 and 527,585 items (2646 unique f/b) were logged between 18:00-20:00 (Fig 4b). For each 2h bin we calculated how many times each unique f/b item was consumed. Food consumption frequency (total logging frequency in 2-hour bins), revealed top-10 items across all 12 bins. Many items appeared repeatedly in top-10 items for each 2h bin, resulting in 28 unique f/b making up the top-10 list in 12 bins. Tea was among the top-10 most frequently consumed items throughout 24 h. Other items remain among the top 10 for one specific window or a prolonged window of time (e.g. coffee from 0:00 to 16:00, eggs from 04:00 to 12:00, beer from 18:00 to 04:00), while others, such as oatmeal, are popular only at one or more bins (Fig 4b).

Out of the 2757 unique f/b, only 788 items (670 foods, 118 beverages) were consumed at least once by >1% of the cohort (~210 participants). Among the rank order of frequently logged items, the top 100 f/b included 86 foods and 14 beverages (Fig 4c and **Table S12**). The most popular item – coffee, a beverage – was consumed by 71.91% of users, while the most popular food – salad – was consumed by 66.69% at least once in 14 days. The 100th most popular item - muffin – was consumed by 12.28% of users at least once in 14 days. Other notable popular items include egg #3 @ 64.01%, chicken # 4 @63.38%, rice #6 @ 52.58%, bread # 9 @45.96%, tea # 13 @ 41.60%, pasta #20 @ 34.09%, beef #39 @ 25.38%, and pork #54 @ 18.53% of users.

At least one of the top 100 popular items was consumed ≥1 times by 99.94 percent of all users and represented 57.01% of all f/b items logged. Due to the over-representation of these items in food records and their consumption by nearly all participants, we focused on their daily consumption pattern. Hierarchical clustering of these top 100 items and their consumption time produced five dominant clusters (Fig 4c, **Figure S5**). For each of these top-100 items, we also calculated their median consumption time (TF50). Cluster 1 (20 items; 12 food and 8 beverages) appeared to be the morning cluster containing several typical breakfast items (e.g. cereal, pancake, oatmeal, bagel, muffin, milk, bacon, blueberry) whose consumption reached TF50 on average by 10:51 (Fig. 4d, e, **Fig S5**). Cluster 2 items (20 items; 17 food and 3 beverages: e.g. bread, butter, peanut butter, yogurt, grape, orange, mango, juice, tea) were consumed almost throughout the day with major peaks around breakfast and lunch, with a minor peak around dinner. These items reached their TF50 at 13:28. Cluster 3 (16 items; 16 food and 0 beverages: e.g. sandwich, hummus, chocolate, spinach) and Cluster 4 (39 items; 39 food and 0 beverage; e.g. pasta, spaghetti, noodle, burger, rice, curry, taco, fish, steak, pizza) were mostly consumed at lunch and dinner. Cluster 3 was slightly more popular around lunch and reached TF50 at 14:50 while Cluster 4 was dominant over cluster 3 at dinner time and reached TF50 around 16:46. Cluster 5 was the smallest one with 5 items (popcorn, beer, ice cream, red wine, and wine), whose consumption started rising at lunch reaching TF50 at 18:48 and reaching peak after all other clusters peaked.

**Fig. 5.**
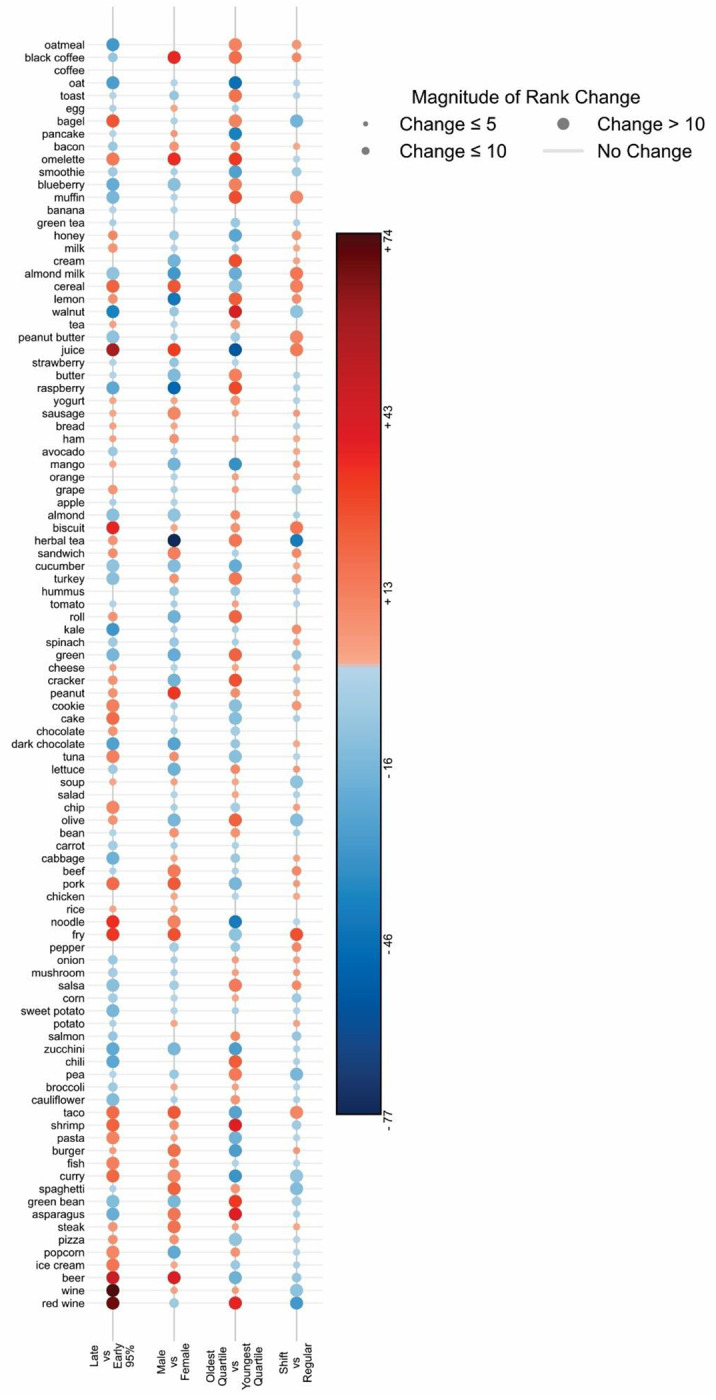
Change in rank of top 100 items between groups (bottom labels). For each pair of compared groups, items climbing up in rank or preferred by group 1 are in blue circles and those sliding in rank or less preferred are represented in red circles. Size The size of the circle and intensity of color represents represent the magnitude of change.

While this rank order of 100 food items was determined for the entire cohort, we asked if food popularity is affected by sex, age work schedule, and preferred timing of eating. To quantify overall differences in the preference of these top items between subgroups, we computed Jensen-Shannon distance (JSD), which measures the dissimilarity between two probability distributions. Specifically, we calculated the JSD between subgroup-level item popularity distributions, defined as the percentage of users in each subgroup that logged each item. Although the observed JSDs were modest (male vs female: 0.07, early vs late eaters: 0.07, youngest vs oldest: 0.07, shift vs standard work schedule: 0.03), all exceeded chance expectations in permutation testing (1000 permutations, p = 0.001). As a global summary measure, JSD reflects aggregate dissimilarity but does not reveal which specific foods or beverages differ in prevalence between groups. Thus, we interrogated the direction and magnitude of change of rank order (upward increase in rank order = more preferred) (Fig 5, **Table S13**). While some of the top 100 items were less affected by these factors, showing minimal rank order change (e.g. coffee, bread, rice, chicken), a subset-including cereal, juice, tacos, and fries shifted substantially (>10) between groups. Young vs. old showed the greatest number of such differences (47 items), followed by early vs. late eaters (44 items), male vs. female (37 items), and shift vs. regular workers (19 items). Group specific preferences included almond milk, dark chocolate, and herbal tea among females, and black coffee, beer, and steak among males. This observed gender difference is comparable to what was reported earlier ^58^. The youngest quartile favored pancakes, burgers, and juices, while older adults preferred red wine, walnuts, and crackers. Shift workers (all types, n = 1541) preferred fries, cereal, and almond milk, though with typically lower rank shifts than other group comparisons. We also found that early eaters—those who ended their 95% eating window before 19:00 (n = 1860 users) prefer walnuts, dark chocolate, and oatmeal, while those 95% eating window ended after 22:00 (n=6278 users) prefer beer, wine, and cereal. Interestingly, all cluster 5 foods and beverages (popcorn, ice cream, beer, wine, and red wine) ranked higher among late eaters.

**Fig. 6.**
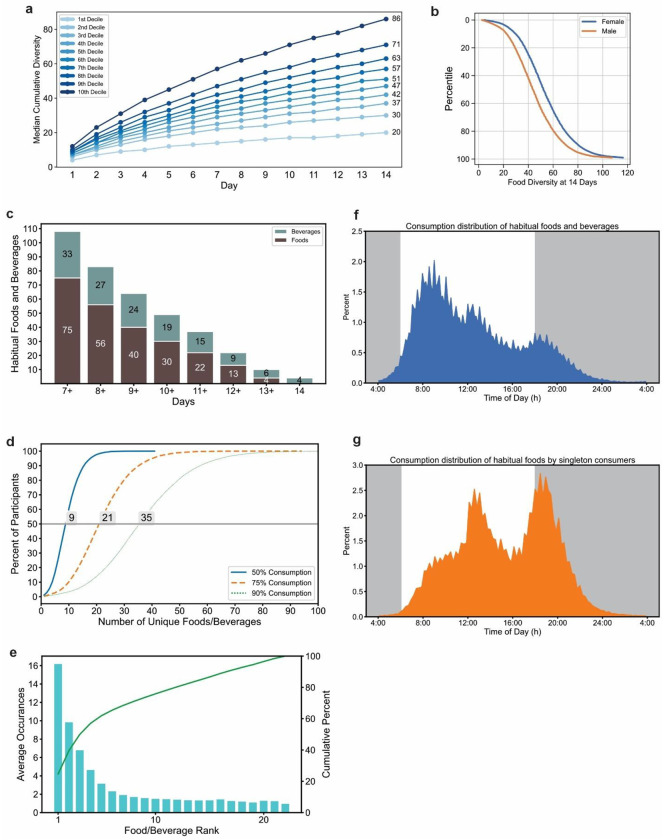
Diversity of foods consumed by individuals including habitual and novel foods. **a**. The cumulative mean number of unique food and beverage items consumed by each decile (~2100 users/decile) over 14 days. **b**. The effects of sex on food diversity. **c**. Number of food and beverages consumed by any 7 or more days by at least ~0.5% or >100 users **d**. mean number of items that constitute 50%, 75% or 90% of all records logged by a user. **e**. mean number of times the highly preferred items are consumed by users in 14 days. Note, the 22nd item onward is almost always consumed only once (novel items). Frequency distribution of the timing of **f**. habitually consumed food and beverages or **g**. novel items shows habitual items are preferred earlier while novel items are more likely consumed at later time in the 24h day.

The sub-group level differences in preferences for top-100 items implied individuals can differ in the diverse types of foods they eat and how frequently or regularly they eat these items. We considered the number of unique f/b consumed at least once during the 14-day period as the food diversity of each individual (20,982 participants after excluding participants who frequently used generic terms such as “breakfast”, “lunch”, “dinner”, “snack” in food logs and excluding common cooking ingredients and additives including oils, condiments, and sauces). From the day-1 of food logs (roughly comparable to one 24HDR) the average food diversity was 9.7 unique f/b (sd 4.74). The cumulative food diversity increased with each additional day of logging reaching a mean of 33.84 unique f/b (sd 13.47) at the end of 7 days and a mean of 50.32 unique f/b (sd 20.21) at the end of 14 days. The bottom decile consumed 20 unique items and the top decile consumed 86 unique items (Fig 6a, **Table S14**). Next, we tested if age, gender, work type or eating window affected food diversity. Food diversity was comparable between the bottom decile for 95%ile eating window (mean items 48.21 sd 20.34; eating window 10h 54m) and the top decile (mean items 48.31 sd 19.39; eating window 16h 0m). Food diversity was not affected much by age (<40y: mean 49.82 sd 21.32, 40-60y: mean 50.73 sd 19.74, >60: 50.09 sd 18.45) or work schedule (ranges from 48.13 to 51.34), but was markedly different between males and females with females consuming almost 10 additional unique items than males (female: mean 53.45 sd 20.12, male: mean 44.88 sd 19.18) (Figure 6b, **Table S15**).

Although an individual may consume a wide diversity of foods or beverages, only a few may be consumed regularly. To assess this, we asked which f/b were consumed by at least 100 people (~0.5% of the cohort) for 14 days. No food was consumed by at least 0.5% of the cohort for all 14 days. Only 4 beverages were consumed by at least 100 people for all 14 days. Coffee was logged by 1534 users for all 14 days; milk, black coffee, and tea are the other 3 beverages consumed by at least 100 users for 14 days. After relaxing the criteria of regular consumption, we found 108 items (75 foods and 33 beverages) were consumed by at least 100 people for ≥7 days (Fig 6c, **Table S16**). For example, the two most consistent items, coffee and tea, were consumed by 10,282 and 2,516 users for ≥7 days, respectively. As we have seen each item has a typical median consumption time (TF50), we asked for a given item if the TF50 for regular consumers (reported ≥7days) is different from that of occasional consumers. Surprisingly, we found for all 108 items, the TF50 from regular consumers is earlier than the TF50 of occasional consumers (reported ≤2days). For example, the TF50 of orange juice for regular consumers was 09:25 while it was 12:15 for irregular consumers. Other examples milk (10:40, 13:20), black tea (10:57, 12:57), sandwich (12:15, 13:50), kombucha (13:54, 15:34), pasta (14:37, 17:57).

**Fig.7.**
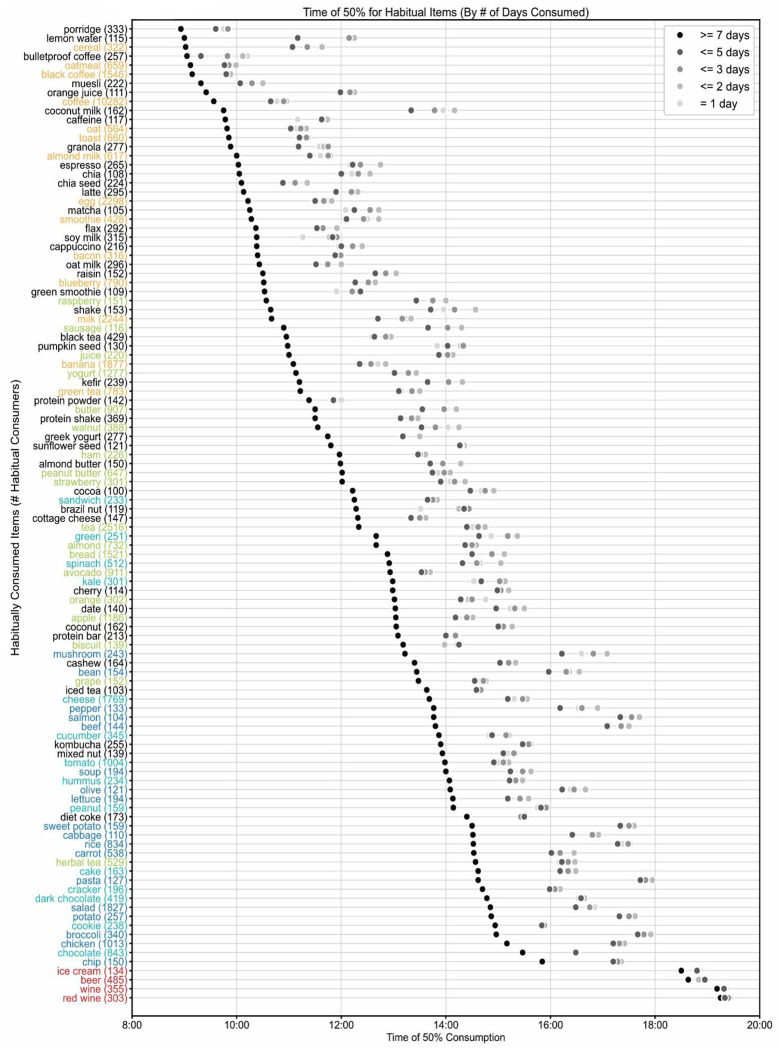
Early consumption of habitual items. Median time of consumption of each of the 108 habitually consumed items. Name of the item and the number of participants who consumed it habitually (at least 10 participants with ≥7d of consumption out of 14 reporting days) and the median time of consumption (TF50) in black circles are shown. Names of items are color coded if they were among the top 100 items in Figure 4. The median time of consumption of the same items when consumed by at least 100 participants only once, ≤2, ≤3, ≤5 days out of 14 days are shown in grey. Notice the black circles for most items are earlier than the grey circles.

As individuals can consume more than one item habitually, we found the average number of food and beverages consumed for ≥7 days was 3.85 items (sd 2.96). In other words, only ~4 out of ~51 unique items consumed in 14 days were consumed for ≥7days. This approach does not account for whether the item is consumed exactly once/day for ≥7 days or more frequently on some days. On average, a user’s most frequently consumed item is logged 16.23 times, or the most preferred item is found in 16 logs. About half a user’s unique items (mean 25.66, sd 11.23) are found in more than one log and the rest of the unique items were found only once in their logs. Dense ranking reveals that any items ranked beyond 22nd in frequency of consumption are consumed exactly once (sd 0) (Fig 6e). This implies that a small subset of items typically accounts for a large share of an individual’s intake. We found for 50% of users (out of the 21k) only 9 unique items are found in 50% of their logs. Similarly, 21 and 35 unique items are found in 75% and 90% of their logs respectively (Fig 6d and **Table S4**). Frequency distribution of time of consumption of these novel foods (consumed only once) and habitual foods revealed that the novel consumption of foods was less likely to occur earlier in the day. As the day goes on, people are more likely to seek novel food and beverages (Fig 6.f, g, Fig. 7).

## Discussion

Temporal eating patterns have recently gained attention, with both perspective and intervention studies demonstrating the potential of such intervention in improving health. This has raised interest in the collection of longitudinal data ^25^. The gold standard of nutrition research 24HDR continues to yield valuable epidemiological data on nutrition quality and quantity, yet there is both explicit and implicit acknowledgement of the need for multiday data collection which faces significant logistics issues for scalability. With the increasing use of smartphones, smartphone-based nutrition monitoring and intervention are on the rise, with almost all of them focused on optimizing nutrition quality and quantity. However, we developed the smartphone app myCircadianClock for monitoring habitual or interventional daily eating pattern.

This study presents data from >20,000 adults who used the mCC app over a 10–14-day period, yielding one of the largest self-reported longitudinal datasets of eating behavior to date. More than 93% of logs were recorded in the phone in real time. The dataset enables fine-grained analysis of temporal eating behavior and food preferences at both individual and population levels. At a population level, several metrics derived from this dataset align with those from large-scale surveys such as NHANES, including the average number of daily eating occasions and the timing of caloric intake. Notably, the dataset also allows the presentation of these metrics across percentile deciles, offering deeper insight into intra-population variability.

We present the temporal eating pattern with various metrices. The median time of all food log (TF50) of an individual represents the midpoint of eating occasions but does not necessarily represent the median time of energy intake. Median time of energy intake has been used to describe early or late eating behaviors ^52,53,59^, but does not report when the eating begins or ends. The average daily window which averages the timespan between the first and last eating episodes is typically reported in many chrononutrition studies ^29,32^. However, day-to-day variation in first or last episodes can result in an artefactually short average window that does not account for ~25% of all eating occasions (**Fig. S1e**). The 95%ile eating window first described by our group ^37^ - by definition – ignores only 5% of early and late eating occasions, but it also requires multiple days of dietary data and typically reports a eating window that is longer than what is reported by average window. By this measure, less than 24% of this cohort have an eating window of <12h (Fig. 1f and **Table S6**), while by average eating window, more than 80% of the cohort eats within ≤12h (**Fig. S1c**). Neither of these two windows report the day-to-day variations in the first and last eating occasions, which are better described by the average first meal shift and average last meal shift ^54^. These two metrices revealed only 1 in 20 participants maintain a consistent eating window where the mean timing of first calorie or last calorie shift is <1h over 2 weeks.

We also found an important relation between eating window and time of day. Irrespective of when the eating window begins, the eating window for >50% of users ended after 20:00 (Fig. 1h), which is comparable to an earlier observation ^60^. This tendency to consume food in the evening may reflect both circadian drive for evening hunger ^61^ and ancestral habit of socializing around food after the end of the day ^62^. Accordingly, those who begin their eating window earlier in the day are likely to have a longer eating window while those who start later have a shorter window that also ends after 20:00.

We also found important relations among food diversity, habitual foods, and time of consumption. There is no universally accepted method to describe food diversity, consistency or regularity of food choices. One of the early methods of food diversity classified foods reported in a 24HR into five major food groups and assigned one point to each food group resulting in a maximum score of 5 ^13^. However, complex foods containing multiple components makes this approach of oversimplifying foods into 5 groups challenging. Hence, other methods have classified foods into larger number of groups and derived indices of healthy eating ^15–17,63–67^. Heterogeneity of such classification systems has made it difficult to compare food diversity between studies. Our approach was to classify foods into usual food names that individuals can relate to. We find food diversity continue to expand from <10 on day 1 to 51 unique foods and beverages in 2 weeks. No single food item was habitually consumed everyday over 14 days. But only a small number of foods were frequently logged; nine items were found in >50% of food records in 50% of users. Habitual foods were more likely to be consumed earlier in the day and as the day progressed the consumption of novel foods (items logged for <7 days) increased.

Observed temporal aspect of consumption of popular foods and of habitual vs. novel foods can also interact with habitual eating windows, such that eating within a given time window may affect food preference. For example, a short eating window as in TRE that ends early or late in the evening can result in different food choices. Early TRE in which eating window ends earlier is sometimes considered better as it aligns the eating window with increased insulin sensitivity early in the day ^68^. However, adopting an early TRE as opposed to late TRE may also benefit from inadvertent improvement in diet quality. Adopting a late TRE can increase the likelihood of consumption of alcohol and energy-dense foods such as ice cream, in the late evening. In support of this, a significant reduction in alcohol intake was noted in a TRE study on firefighters ^41^.

We did not collect comprehensive health information nor estimated energy intake as are collected under NHANES. Hence, we cannot make any definitive conclusion about temporal eating patterns and health. However, the dataset sheds important insights into how age, gender and work type affects food timing and food preferences. The working population of most countries including the US have ~20% shift workers who can have different eating time or food preference than the regular workers. Some studies have reported ~20% of NHANES cohort are shift workers ^69^, very few studies compare the food habits of shift workers against regular workers ^69–71^. We found the shift workers were more likely to have a longer eating window and have more irregular first and last meal shifts. Yet, the data also shows at least 20% of shift workers have a 95%ile eating window of <12h (**Table S6**). We also found differences in eating patterns between sexes or between young and old participants, but these differences were smaller compared to that between shift and regular workers. For food diversity, the gender difference was larger than any other parameter; even participants who ate their food within a shorter or longer window had comparable food diversity. Such increased preference for diverse foods by females may reflect the known shopping habits of females preferring to purchase more diverse foods ^72^. As more than 50% of the unique food items were consumed only once in 14 days, the food preferences of each subgroup are better revealed by the rank order of the top 100 frequently consumed items. In this metric, the rank order changed for almost 50% of items (47out of 100) between young and old and relatively less by gender or shift work (37 out of 100 and 19 out of 100, respectively). In summary, the data also suggests that as one goes through life and chooses different types of work, what and when or how consistently one eats can also change. Furthermore, nearly 20% of adults at any given time adopt dietary intervention plan ^73^ by changing what, how much or when to eat. All of these factors need consideration while making correlation between dietary habits and current health status or prediction on future health status based on current dietary habits. Conversely the plasticity of dietary habits also offer an avenue to develop intervention plans to improve health.

This data set is consistent with previous findings in much smaller data sets regarding the 95% eating window duration ^37^ and meal frequency from NHANES data based on 2 days of dietary recall ^74^. However, due to the large sample size (n >20,000), real-time logging (84.43% within an hour and 99.63% within 24 hours), and longer eating assessment (10-14 days), it can uncover novel insights into temporal eating patterns.

### Limitations:

The cohort is a self-selected group who voluntarily used the app. They are more likely to be higher educated, English speakers. The food logs did not have portion size information, and hence, the dataset lacks total energy intake information. Additionally, food diversity may be influenced by participants’ food knowledge, their ability to disaggregate complex dishes (e.g. salads), and social desirability bias in reporting. Given the name of the app, the users may also be aware of circadian rhythms in sleep-wake and feeding-fasting cycles and may have modified their eating pattern to have a shorter eating window than they had prior to use of the app. As with any self reported timing of food intake, we cannot ensure that the time of intake reported is actual time of consumption. In the absence of their habitual sleep time, sleep quality, and physical activity pattern, it is difficult to fully interpret the physiological significance of eating time on health. Relatedly, the definition of daily activity taking place on a 04:00 to 3:59:59 basis was chosen to account for average human behavior, yet some individuals such as night shift workers who have different sleep times may be incorrectly characterized by this choice.

## Conclusion

This large-scale study that captured 10-14 days of participant dietary intake in real time found that both the timing and the nutritional components of dietary intake are highly variable. However, this method is also impractical for the collection of nutrition quantity at scale. Thus, newer approaches are needed that combine longitudinal food records along with randomly selected a few days of in-depth reporting of portion size and detail food description to better assess dietary patterns from individuals to population.

## Methods.

### Study overview.

This exploratory observational study was approved by the Salk Institute Institutional Review Board (15-0003). All dietary intake data were collected through the myCircadianClock (mCC app). The mCC app is a non-commercial research smartphone application designed to primarily collect the timing of dietary intake over several days or weeks ^36^. It is available free of charge for all Android and iPhone smartphones. The app is designed to be used in multiple studies with study-specific and user specific customization. Specific custom versions of this app have been used in several lifestyle intervention studies ^38–45,75,76^ where participants meeting enrolment criteria used the app after in-person consent for the respective studies. For this study, the app was customized for web-based or in-app consent to collect dietary intake information from large number of study participants.

During the app onboarding steps, user data (age, sex, education level, work type) were collected. Participants were asked to log their current lifestyle for 2 weeks. They received an automated daily reminder to log their dietary intake. During these two weeks, participants could not review their previous entries to minimize potential influence of reviewing records of dietary intake or timing on subsequent nutrition intake. At the end of the two weeks, they could see all their entries and their calculated 95%ile eating window. Hence, any data logged after the initial 2 weeks were likely influenced by the dietary feedback and were not used in the current analysis.

### Participants.

Participants were recruited from January 9th, 2015 to February 19th, 2024. Adults (at least 18 years old), who could comprehend consent and adhere to recording daily nutrient consumption in English were included. There were no geographical restrictions on participation. Participants’ characteristics are in Table S1. Participants were recruited using public tools such as flyers, social media posts, and announcements of the study during public speaking opportunities. Participants registered to participate on the myCircadianClock website (www.mycircadianclock.org) or after downloading the app. During the registration process, participants were provided with an electronic informed consent. Upon consenting to participate, a user specific access code for the mCC app was provided to activate the app on their smartphone.

### Data Collection.

Data for this analysis was collected over a 2-week period to assess current lifestyle with primary focus on eating patterns. Participants were asked to log all dietary intake with options to also log water, medications, sleep, exercise, and health metrics.

There were three types of food and beverage logs. Participants were asked to log their dietary intake at the beginning of the consumption event, by (a) taking a picture of the item and providing a description of the food or beverage or (b) logging the description of the food or beverage without a picture. If they could not log at the time of consumption because they forgot or due to social setting where phone use may not be appropriate for the user, they could (c) enter the description of the food or beverage and indicate the time and date of consumption. If the user’s phone did not have internet access at the time of recording, the time-stamped log waited in a queue until internet could be accessed. All information was encrypted during transmission from the phone to the server. The time when the log reached the server was also recorded. The local time on the phone at the time of consumption (not the time of logging as in scenario c nor the time of recording in the server) was used in all nutrition timing reported in this manuscript. Once logged, an item could not be deleted and the timing could not be changed.

### Data Quality Control.

To be eligible for analysis, participants were required to meet logging compliance criteria for at least ten of their first fourteen days using the mCC app. A logging day is considered compliant if it consists of two or more calorie-containing (water and non-caloric beverages excluded) eating occasions at least five hours apart. Logging occasions were considered to belong to the same ‘compliance’ day for compliance calculation, beginning at 04:00 on one calendar day and ending at 03:59:59 the following calendar day. The log time used for compliance and analysis often differed from the server’s recorded log time. This occurred when the user forgot to log or could not log at time of ingestion because of social circumstances. It also occurs when the user’s phone does not have internet access—logs are then queued to be sent upon restoration of internet access. We consider any log with greater than fifteen minutes of difference of time between server recorded reception time and user recorded ingestion time to be a ‘delayed log’. 15.57% of logs are delayed logs. To distinguish between delayed logs caused by lack of internet access (which would still reflect accurate ingestion time) and ‘backlogs’ (logs where the user relies on memory to approximate ingestion time), we examined the distribution of minutes for all delayed logs and found that minutes that are multiples of five (:00, :05, :15, etc.) exhibited preferential logging. A chi-square test comparing the minute-level distribution of delayed versus real-time logs revealed a statistically significant difference between the two (χ^2^ = 59,298.74, df = 59, p≅0.0). Therefore, we considered any ‘delayed’ log occurring at minutes with large differences in logging frequency from expectation a ‘backlog’. 41.49% of delayed logs are backlogs, accounting for a total of 6.46% of all data. To further verify data quality, we compared the measured statistics of the entire cohort against the remaining cohort after removing the 163 users with only delayed logs. We observed very marginal differences in the distribution of eating window metrics between the two cohorts.

Similarly, metrics that deal with food diversity or popularity exclude the following non-descriptive phrases that we observed to be frequently logged: ‘dinner’, ‘breakfast’, ‘lunch’, ‘snack’, ‘meat’, ‘protein’, ‘fruit’, ‘berry’, ‘seed’, ‘vegetable’, ‘appetizer’, ‘brunch’, ‘dessert’, ‘meal’, ‘leftover’ and ‘nut’. We examined the impact of disallowing non-descriptive food and beverage phrases for meeting compliance criteria and found that 876 users lost compliance without the allowance of non-descriptive phrases. We additionally looked at excluding days where the user was found to be compliant off of a single unique item (e.g., all logs for a day are the phrase ‘meals’). We compared excluding the 876 non-descriptive users and the additional 103 single-item users against the entire cohort and again found very marginal differences in the distribution of eating window metrics

### Statistical Methods.

All statistical tests were conducted on distinct samples. All tests, with the exception of permutation tests of Jensen-Shannon distance, were two-tailed. Kolmogorov-Smirnov and Levene’s tests were performed to test data for normality before selecting non-parametric tests. After non-parametric tests with multiple comparisons, post-hoc pairwise between-group comparisons were performed using Dunn’s test as implemented in the scikit-posthocs Python package, which outputs a p-value but does not yield a test statistic. All other statistical tests report both test-statistic and p-value when appropriate. Effect sizes for non-parametric between group-comparisons were calculated as Cliff’s delta. Wilcoxon signed rank test was used for within-group comparisons and paired with Rank-Biserial as its effect size calculation.

Chi-squared tests of independence were used to assess associations between decile membership for eating window related-metrics and demographic qualities.

Permutation testing was conducted for Jensen–Shannon distance using n = 1,000 permutations to determine whether the observed distance between subgroup item popularity distributions was significantly greater than expected by chance.

Exact p-values are reported unless they are below the machine precision limit, in which case they are denoted as p≅ ~0.0.

### Item Parsing.

The food parser is an automatic text processing pipeline designed to extract meaningful food, beverage, and medication-related items from mCC’s mobile app-based food logs. The software for log parsing was coded in Python with extensive use of NLTK for language processing. mCC app logs consist of one or more food, beverage, or medication-related words or phrases written with the user’s natural language description. Individual food items or phrases within the same log are expected to be separated by a comma. Therefore, each log is split at its commas into multiple ‘sub-logs’, which are individually pre-processed by lowercasing and removing all special characters and punctuation. Commonly used English words (e.g., ‘the’, ‘a’, ‘is’, etc.) and any numbers not immediately preceded or followed by a letter were also removed. Phrases that indicate amounts of measurements (e.g., ‘oz’) are also removed. Any remaining words within each sub-log are converted to their root forms (e.g. ‘eggs’ to ‘egg’). These words are compared against a dictionary of common food-related misspellings and abbreviations for spell correction. Instances where known words were incorrectly joined were also identified and separated (e.g., blueberrymuffin to blueberry muffin). The resulting processed sub-logs were then matched to our food item dictionary based on phrase length to find individual items within each sub-log (e.g., single-word sub-logs were checked against single-word dictionary entries). Sub-logs were matched with food-related phrases of up to five words long, based on the total length of the sub-log. If no recognized phrases of that length were found, subsets of the sub-log were compared against dictionary items of increasingly shorter lengths.

### Food Dictionaries.

The food parser relies on the usage of two custom dictionaries: a food item dictionary and a typo dictionary. Users enter text descriptions of their food and beverage consumption. Common typos and abbreviations are changed into the assumed intention (e.g., “coffeee” to “coffee”) using a typo dictionary. Next text descriptions are regularized into comma separated lists of components using a food dictionary (e.g., “Spaghetti,Salmon,Melted Cheese,Paprika” to “spaghetti”, “salmon” “cheese”, and “paprika”).

The typo dictionary consists of 2,336 common typos and abbreviations of items found in the food item dictionary.

The food item dictionary contains 5396 phrases (consisting of 1-5 words), split into seven types: ‘foods’ (2285), ‘beverages’ (542), ‘modifiers’ (964), ‘stopwords’ (547), ‘water’ (35), ‘medication’ (1016), and ‘selfcare’ (7). Foods and beverages are individual descriptors of food and beverage items (e.g., ‘muffin’ or ‘orange juice’). Modifiers and stopwords include adjectives and other modifying phrases (e.g. ‘freshly squeezed’, ‘without sugar’) or unrelated words commonly found in food descriptions (e.g., ‘nothing’, ‘eat’). Water includes water-related branding and phrases (e.g., ‘dasani’, ‘tap water’). Medication includes medication and supplement-related terms (e.g., ‘vitamin c’).

The typo and food dictionaries affect the outcomes of downstream analysis. The exact choices of regularizing food description here will directly affect measurements of food diversity and popularity.

### Logs and Eating Events.

A participant log is any distinct entry made by an mCC app user recorded by our server. Logs are required to have a natural language description of the user’s intake for that specific log. No constraints on the style of description or number of items within a log are made. Logs are assigned an original ‘log type’ (food, medication, beverage, or water) by the user, indicating the majority of item types within a log. An ‘item’ is a singular, food parser-recognized word or phrase (e.g., ‘hot dog’). Individual items are re-assigned an ‘item type’ based on our dictionary (e.g., ‘avocado’ would be assigned ‘food’ regardless of the type of the log it is found in). A ‘caloric item’ is any recognized item that is designated as a ‘food’ or ‘beverage’. Water, club soda, seltzer, and variants of sparkling water are considered ‘water’ and not a ‘beverage’. Non-caloric teas and black coffee are considered ‘beverages’ due to their impact on the fasting state. Any food or beverage items within logs made by the same participant without a 15-minute or greater interval between log entry times are part of the same ‘eating event’. For example, caloric items within logs at 09:45, 09:56, and 10:10 belong to the same eating occasion, but additional items within a log at 10:26 would be counted as part of a separate eating occasion. Water and other non-caloric items are not counted in eating events. Eating events are only calculated from logs within the same compliant day, beginning at 04:00 on the first calendar day and ending at 03:59:59 on the following calendar day. Definition of various metrices used to describe temporal eating patterns or regularity of foods are describes below.

### Time to 50% Caloric Log.

The time of 50% of logging is calculated as the median log time for all food and beverage logs (excluding water) across the entire 14-day period.

### Time to 50% for Food and Beverages.

The time of 50% for a specific food or beverage is calculated as the median log time for that specific food or beverage across the entire 14-day period.

### Average Eating Window.

The average eating window is measured by calculating the average time between the earliest and latest caloric logs for each day on which the participant logged at least two caloric logs, with a minimum of 5 hours between first and last caloric log.

### 95% Eating Window.

The mid 95%ile eating window is calculated as the time window in which 95% of all caloric (food or beverage) items derived from all mCC logs were entered ^37^. The earliest and latest 2.5% of caloric items are removed. Medication, water, and other non-food/non-beverage descriptors are not included in calculating eating windows.

### Intake Irregularity (First and Last Eating Occasion Shift).

The average shift in first and last caloric intake times measures the irregularity of a participant’s food habits. Intake shift was measured for the timing of both the first and last caloric events on each day where the participant logged at least two caloric logs, with a minimum of 5 hours between first and last caloric log. Each measurement of shift is the absolute difference in time between a specific intake event occurring on successive days ^54^. The first caloric intake shift is the shift in the timing of the first calorie consumed during the day, while the last caloric intake shift measures the shift of the last caloric intake event during the day.

### Food Diversity and Popularity.

Food diversity is measured as the diversity of dictionary-matched caloric items logged by the participant. It excludes multiple common non-descriptive phrases: ‘dinner’, ‘breakfast’, ‘lunch’, ‘snack’, ‘meat’, ‘protein’, ‘fruit’, ‘berry’, ‘seed’, ‘vegetable’, ‘appetizer’, ‘brunch’, ‘dessert’, ‘meal’, ‘leftover’ and ‘nut’. Measurable food diversity may be impacted by a user’s preferred level of descriptiveness for their logged items and their ability or willingness to disassemble food items (e.g. ‘salad’) into components and ingredients. As such, 141 ingredient-like items including condiments, sauces, and common cooking ingredients (e.g., ‘salt’, ‘oyster sauce’) are not included in diversity calculations. Although user logging style is not controlled, consistency in verbosity is encouraged–users can re-select the exact descriptors they have used previously when re-logging items. Food popularity is measured as the percentage of users that log an individual food or beverage item at least once.

### Habitual Food.

A ‘habitual food’ is a food or beverage consumed by at least 100 participants (~0.5% of the cohort) for at least seven out of fourteen days.

## Supplementary Material

This is a list of supplementary files associated with this preprint. Click to download.

• TableS13.xlsx

• TableS12.xlsx

• SupplementaryMaterials.docx

• TableS4.xlsx
